# Assessing the Student Nurses’ Knowledge of Oral Health Care

**DOI:** 10.3390/nursrep13030097

**Published:** 2023-08-18

**Authors:** Gemma Marquès-Pellejà, Marta Roqueta-Vall-llosera, David Cámara-Liebana, Susana Mantas-Jiménez, Sandra Gelabert-Vilella, Alícia Baltasar-Bagué, Glòria Reig-Garcia

**Affiliations:** 1 Serveis de Salut Integrals Baix Empordà, 17230 Girona, Spain; gmarques@ssibe.cat; 2 Department of Nursing, Faculty of Nursing, University of Girona, C/Emili Grahit, 77, 17003 Girona, Spain; david.camara@udg.edu (D.C.-L.); susana.mantas@udg.edu (S.M.-J.); sandra.gelabert@udg.edu (S.G.-V.); alicia.baltasar@udg.edu (A.B.-B.); gloria.reig@udg.edu (G.R.-G.); 3 Health Gender and Aging Research Group, University of Girona, 17003 Girona, Spain; 4 Quality of Life Institute Research Group, University of Girona, 17003 Girona, Spain; 5 Health and Health Care Research Group, University of Girona, 17003 Girona, Spain

**Keywords:** nursing, oral health, nursing students, health promotion

## Abstract

Oral health is crucial for the overall well-being and quality of life, and nurses play a significant role in promoting it. This study assessed the oral health knowledge of fourth-grade nursing degree students. Seventy-two students completed a questionnaire covering sociodemographic variables, oral health-related factors, knowledge about oral health, and perceptions of its importance and learning experiences. The results showed that 83.3% of students attended regular dental check-ups for preventive purposes, and 55.6% had visited a dentist during the last year. Most of the students reported experiencing cavities (66.7%) and undergoing orthodontic treatment (54.2%). The average knowledge score in oral health was 6.4 out of 10, and students recognized the importance of oral health in the nursing role. However, their knowledge acquired during their nursing degree scored relatively low, being 2.5 out of 5. Notably, students who valued problem-based learning achieved higher knowledge scores (*p* < 0.05). Overall, fourth-grade nursing students demonstrated a moderate level of oral health knowledge. Improving oral health education within nursing curricula, particularly through problem-based learning, is essential to enhance their preparedness in addressing oral health issues effectively. This study was not registered.

## 1. Introduction

The World Health Organization (WHO) defines oral health as “the state of the mouth, teeth, and surrounding tissues that enables individuals to perform basic functions such as eating, speaking, and smiling, and affects psychosocial aspects such as self-confidence, well-being, and the ability to socialize and work without pain, discomfort, or embarrassment”. Oral health is a fundamental indicator of overall health, well-being, and quality of life [1]. It encompasses a range of diseases and conditions, including dental caries, periodontal (gum) disease, tooth loss, oral cancer, and other less common oral diseases [2]. These oral health problems pose a significant burden on the healthcare sector in many countries, causing pain, discomfort, deformities, and even mortality [3]. According to the most recent global burden of disease study, oral diseases are highly prevalent worldwide, affecting 3.5 billion people [4].

NICE guidelines indicate that certain groups of people, such as those with diabetes, cardiovascular diseases, pregnancy, menopause, smoking habits, and different socioeconomic backgrounds, have specific dental needs [5,6]. However, recommendations to address the burden of oral diseases have emphasized the importance of population-wide approaches [7]. According to NICE [6], local authorities and partners should incorporate oral health promotion into existing services for all children, young people and adults. Regarding young people, there is a specific recommendation to promote a whole-school approach to oral health among high school students. Oral health during childhood and adolescence is crucial for adult life, and in this regard, Spain has developed the Child Dental Health Plan with the aim to improve oral health, promote healthy eating habits to prevent dental issues, and enhance access to dental health services for children and young people [8].

Typically, oral health care is primarily provided by dentists and dental care professionals [5]. In Spain, this attention includes diagnostic, therapeutic, and preventive activities, as well as health promotion and education focused on oral health. However, certain treatments such as the restorative treatment of primary teeth, orthodontic treatments, extraction of healthy teeth, and the performance of complementary tests may not be covered [1]. Therefore, not all individuals have equal access to oral care, particularly those with low income or without private health insurance [9,10,11], to whom, according to Braveman [12], disparities in oral health care are unnecessary, avoidable, and considered unfair and unjust. One of the barriers to equality in oral health care is the lack of attention given to oral health by non-dental healthcare professionals, such as nurses, pharmacists, physicians, and physician assistants [13]. Therefore, it is crucial for health opinion leaders, including nurses and general practitioners, to reinforce oral health campaign messages. Future oral public health endeavors should engage a wider range of community influencers and utilize social networks [14]. However, some studies have identified certain inadequate nursing standards of practice for oral health care [15,16]. Moreover, other authors have suggested that there is a high prevalence of poor knowledge and negative attitude toward oral health among nursing students [17,18]. Therefore, nursing programs should equip graduates with core competencies to identify oral disease risks, conduct oral examinations, provide oral health information, connect oral health with diet and lifestyle counseling, and make referrals to dental professionals.

An appropriate training and encouragement for the promotion of oral health might provide suitable care for the prevention of dental diseases, and in consequence, it should be included in the nursing training curricula [17] for achieving better results in oral health [17,19].

The Spanish healthcare workforce comprises over 316,094 registered nurses [20] who, if adequately educated and trained in oral health, would have had a potential impact on improving access and quality in this health dimension.

However, in Spain, most nursing degrees only superficially address oral health, lacking the in-depth focus given to other diseases [21,22,23,24]. For instance, the University of Girona Nursing Faculty includes content on oral health within two subjects: ‘Healthy Person Throughout Life’ and ‘Community Nursing I,’ essentially from a preventive health perspective [21]. While some studies have assessed the attitudes and practices of nurses concerning oral health [25,26,27], to the best of our knowledge, no existing studies have evaluated the knowledge of future nurses on oral health.

As a concerning phenomenon that can have negative repercussions on the overall health care of patients, the existing lack of knowledge about oral health among nurses should be taken into consideration. Although during the nursing degree, competencies, attitudes, and knowledge are assessed on many essential subjects for the profession [25,26,27], there are no specific outcomes regarding the knowledge of future nurses in oral health. Hence, the aim of the present study is to analyze the knowledge of oral health among the final-year students of the nursing degree. Identifying the knowledge of future nurses in oral health and determining their readiness to provide optimal oral health care to the community is essential for formulating effective education and awareness strategies within the nursing profession. This may encompass educational programs, workshops, and resources aimed at enhancing the understanding and application of sound oral health practices during their nursing education and in their practical healthcare settings. Ultimately, a more robust integration of oral health into nursing educational programs has the potential to significantly contribute to the overall enhancement of health outcomes for both individuals and communities.

## 2. Methods

### 2.1. Design

This is a cross-sectional study of fourth-grade nursing degree students on their oral health knowledge acquired.

### 2.2. Sample

The study recruited a total of 139 participants, which represented 100% of the fourth-grade nursing students of the Nursing Faculty at the University of Girona. The students were invited to participate in the study through email contact, where a researcher provided them information about the study and the questionnaire. A distribution list was used for sending the invitation email.

The inclusion criterion was: studying as last-grade students of the nursing degree at Girona University. The contents in oral health are given in different subjects during the four years of the nursing degree and are not limited to a specific course, which is the main reason why we included students in their last year of study so as to be able to evaluate their knowledge of oral health just before finishing their degree. Students who had previously completed the oral hygienist training cycle were excluded.

### 2.3. Instruments

An ad hoc, anonymous and self-administered questionnaire was prepared, consisting of 4 sections. In the first section, sociodemographic variables were collected, such as: age, gender (man, woman, non-binary), place of origin (Catalonia, Spain, outside of Spain), previous education (secondary education, training cycle or university degree), working in the healthcare field (yes or no), employment setting (hospital, primary care, nursing homes, others), and if any family members were employed in the health field (yes or no). The second section included personal variables related to oral health: whether undergoing preventive dentist check-ups (yes or no), frequency of dentist visits (less than once a year, once a year, more than once a year, more than twice a year), any presence of cavities (yes or no), the number of cavities if present (I don’t know, one, between two and five, more than five), and if participant is a carrier of orthodontics (yes or no). The third section consisted of a sixteen-question oral health knowledge test related to the following: general knowledge of oral health (2 questions); knowledge of teething (2 questions); knowledge of dental tissue (1 question); knowledge about aspects of teeth (3 questions); and knowledge of oral health problems’ risk factors and preventive activities (8 questions). Every item was a question with four possible answers where only one was correct. In order to facilitate the final score test interpretation, this was calculated by conducting the mean of the test’s correct answers, whereby 0 points indicated the minimum grade and 10 points indicated the maximum grade. This third section was performed after reading the most recent literature and with the collaboration of experts in the field. Finally, the fourth section of the questionnaire gathered data regarding participants’ perception of importance of oral health and their knowledge acquired during the nursing degree through 9 questions: (1) importance of oral health in overall health, (2) importance of the nursing role in the prevention and promotion of oral health, (3) observation of prevention and promotion activities related to oral health during clinical practices, (4) acquisition of oral health knowledge during the nursing degree, (5) satisfaction with the oral health content received during the nursing degree, (6) satisfaction with the oral health content received through master classes, (7) satisfaction with the oral health content received through seminars, (8) satisfaction with the oral health content received through problem-based learning methodology, and (9) consideration of the need to receive more oral health content in the nursing degree. Every question was measured on a 5-point Likert scale, where 1 represented the lowest score and 5 represented the highest score. Participants had 10 min to complete the questionnaire.

### 2.4. Validity and Reliability

Prior to the study, a pilot test was conducted with 10 students in order to evaluate the performance and reliability of the measurement instrument. All participants responded to the different questions of the questionnaire without any problems and no changes were required. Additionally, we assessed the internal consistency reliability of the 12-question test by calculating Cronbach’s alpha, obtaining a range between 0.72 and 0.84.

### 2.5. Data Collection

For data collection, an anonymous self-administered questionnaire was used. The researchers reached out to 142 nursing students in their final year of nursing degree, inviting them to participate in the study. Each participant received a unique link to access and complete the questionnaire online. It was emphasized that the questionnaire must be completed within a time limit of 10 min, as the link would become unavailable thereafter. The distribution of the link was facilitated through the use of an email distribution list.

### 2.6. Ethical Considerations

This study was approved by the University of Girona’s Ethics Committee (code CEBRU0004-23). The researchers informed all the participants about the objective of the study. The questionnaires were distributed to the participants through a distribution list and the information collected did not contain any personal data that could identify these participants, so anonymity was always maintained. Moreover, participants were informed that their participation did not have any risk. The data were analyzed by a researcher and the principles defined in the Declaration of Helsinki were followed.

### 2.7. Data Analysis

The data obtained from the questionnaires were analyzed using the statistical package IBM SPSS Statistics for Windows version 28.0. Continuous variables were described as the mean and measures of dispersion (standard deviation, median, and interquartile range). Categorical variables were described in terms of absolute frequency and percentage. The bivariate analyses were conducted by *t*-test and ANOVA test.

## 3. Results

The final sample comprised 72 students, representing 50.7% of the total participants. Among them, 84.7% were women. The average age of the students was 22.7 years (SD 2.8). Out of the total sample, 83.4% (*n* = 60) were originally from Spain and studied the nursing degree after completing secondary school (66.7%). A total of 62.5% of the participants had previous experience in the healthcare field, with a significant proportion (43.1%) having worked in a hospital setting. Additionally, 47.2% reported having family members working in healthcare (Table 1).

In terms of oral health, 83.3% of nursing students attended regular dental check-ups for preventive purposes. Furthermore, the majority (55.6%) had visited a dentist within the last year. Among the students, 66.7% reported having experienced cavities at some point in their lives, with 34.7% reporting between two and five cavities. Additionally, 54.2% had undergone orthodontic treatment (Table 2).

The average grade on the test was 6.4 (SD 1.02) points. The lowest grade recorded was 4.38, while the highest grade achieved was 8.75. The questions that had better results were: “The number of teeth sets throughout life” (81.9% correct) and “Knowledge about the most common untreated oral pathology worldwide” (87.5% correct). Lower scores were obtained in the questions related to: “The risk factors for gingivitis” (79.2% incorrect) and the correct answer regarding the amount of toothpaste (68.1% incorrect). Scores of each item of the test are presented in Table 3.

Students who worked in the healthcare field achieved slightly higher grades on the test compared to those who did not (6.5 vs. 6.2, range: 0–10), especially those working in nursing homes (7.1 vs. 6.4, range: 0–10). However, these differences were not found to be statistically significant (*p* > 0.05).

Additionally, no significant differences were observed in test scores based on variables such as having family members working in the healthcare field, receiving preventive dental care, experiencing cavities, or undergoing orthodontic treatment (*p* < 0.05).

The final section of the questionnaire gathered data regarding participants’ perception of the importance of oral health and the knowledge acquired during their nursing degree.

Regarding participants’ perception of the importance of oral health, the variable related to the overall importance of oral health received the highest score of 4.5 (SD 0.6; range: 1–5). The role of nurses in promoting and preventing oral health obtained a score of 4.2 (SD 0.9; range: 1–5). When assessing the knowledge about oral health acquired during their nursing degree, nursing students obtained an average score of 2.5 (SD 0.9; range: 1–5) points. Specifically, they rated the knowledge gained in master classes with an average score of 2.4 (SD 0.9), knowledge acquired in seminars with an average score of 2.3 (SD 0.9), and knowledge obtained through problem-based learning methodology with an average score of 2.7 (SD 0.4; range: 1–5). The overall satisfaction with the knowledge acquired in this topic was rated at 2.4 (SD 0.9; range: 1–5). In terms of experienced oral health interventions during the practical, nursing students assessed it with an average score of 2.8 (SD 1.2; range: 1–5) points. Finally, nursing students perceived that the nursing degree program should include more content on oral health, with an average rating of 4.4 (SD 0.6; range: 1–5) (Table 4).

Regarding sociodemographic variables, male participants scored higher in the variables “Acquisition of oral health knowledge during the nursing degree” (2.80 (0.45) vs. 1.80 (0.87); *p* < 0.017) and “Satisfaction with the oral health content received through master classes” (2.00 (0.00) vs. 1.46 (0.012); *p* = 0.02). Nursing students who had previous experience in the health field expressed a higher need for more oral health content during their nursing degree (1.98 (0.02) vs. 1.86 (0.07); *p* = 0.04). Participants with family members working in the health field perceived the importance of the nursing role in the prevention and promotion of oral health more positively (4.47 (0.12) vs. 3.94 (0.16); *p* = 0.01). No significant differences were observed in oral health attitudes based on the rest of the sociodemographic variables.

Statistically significant differences were not observed in the assessment of different variables related to attitudes based on whether students visited to the dentist preventively (*p* > 0.05) or had worn orthodontics (*p* > 0.05). However, students who had not experienced cavities considered the oral health content during their degree to be more sufficient than those who had experienced cavities (2.29 (0.20) vs. 1.75 (0.12); *p* = 0.03). The rest of the variables did not show significant differences.

We observed a positive association between the exam grade and the overall importance of oral health (6.5 vs. 5.8; range: 1–10; *p*-value > 0.05), even though non-significant, as well as the nursing students’ knowledge about oral health (6.4 vs. 6.1; range: 1–10; *p*-value > 0.05) and the assessment of knowledge acquired during the nursing degree (6.6 vs. 6.4; range: 1–10; *p*-value > 0.05). Conversely, we found a negative, yet non-significant, association between the test grade and the following variables: observing actions promoting oral health during clinical practice (6.4 vs. 6.1; range: 1–10; *p*-value > 0.05), satisfaction with knowledge acquired during the nursing degree (6.6 vs. 5.9; range: 1–10; *p*-value > 0.05), and satisfaction when using master classes (6.5 vs. 6.3; range: 1–10; *p*-value > 0.05) and seminars (6.5 vs. 6.2; range: 1–10; *p*-value > 0.05). However, nursing students who had a higher regard for the knowledge of oral health acquired through problem-based learning methodology achieved significantly better grades on the test (6.5 vs. 5.0; range: 1–10; *p*-value = 0.03). Moreover, students who expressed the belief that the nursing degree should improve its content on oral health also obtained better grades on the test (6.5 vs. 5.3; range: 1–10; *p*-value < 0.02).

## 4. Discussion

The study aimed to assess the knowledge of fourth-grade nursing students regarding oral health and identify their potential impact on this knowledge. Findings revealed that a majority of nursing students regularly attended dental check-ups for preventive purposes. Although the latest Eurobarometer survey [28] showed that 43% of the Spanish population visited a dentist during the last year, previous research in younger individuals has indicated a limited utilization of regular dental check-ups among this group [29].

However, other variables such as being female [30] and possessing high levels of health literacy [31] can influence the adoption of dental preventive measures. In our study, the majority of participants were female in their final year of nursing degree, which could explain the higher rates of dental check-up attendance observed compared to the general population.

Additionally, slightly over a half of the participants reported having experienced cavities at some point in their lives and had received orthodontic treatment. According to Peltzer [32], university students have a high prevalence of poor dental health and engage in unhealthy oral behaviors, which can increase the risk of developing cavities. Moreover, other factors such as poor dietary habits and tobacco consumption [33,34] have been associated with the presence of cavities. Studies conducted by Varela [35] and Yun [36] have found that smoking and an unhealthy diet are significant risk factors among university students.

Although this study did not collect variables related to lifestyle, there is evidence that the consumption of sugars and tobacco in Spanish university students is common. According to the ANIBES [37] study, sugar consumption by this age group is skyrocketing due to the consumption of added sugars such as: sugary drinks, juices, and pastries. In addition, Arjona et al. [38] concluded that 40.9% of Spanish university students have a low adherence to a Mediterranean diet that suggests a high consumption of fats and sugars. Regarding tobacco consumption, 19.9% of university students in Spain reported having consumed it in the last 6 months (20.2% women; 18.9% men) [39]. As Correa states [40], some variables such as gender and smoking environment have a significant association with tobacco consumption.

Therefore, it is essential to emphasize the promotion of oral health education and positive oral care practices within this particular population. Additionally, promoting safe academic environments that are healthy and free of tobacco smoke is essential. Many Spanish university campuses are aligned with this objective as health-promoting agents [41].

Oral care is a fundamental nursing procedure that significantly impacts patient well-being and overall health [42]. As primary healthcare providers, nurses bear the responsibility of providing oral care as part of their duties [43]. Nevertheless, several studies have identified inadequate oral care practices in nursing [15,16], highlighting the need for improvement. According to Dagnew, enhancing nursing curricula to include comprehensive knowledge on oral care could potentially address this issue [44].

Our findings reveal that final-year nursing students possess a moderate level of knowledge in relation to oral health. This is particularly evident among students who are simultaneously pursuing a nursing degree while working in home care nursing. This is understandable as providing oral care to nursing home residents constitutes an integral part of their caregiving routines [45]. Those students who had experienced cavities or went to the dentist preventively did not demonstrate higher levels of oral health knowledge. Thus, it appears that personal experience or interest in oral health might not have influenced their knowledge. In contrast, a previous study among nurses revealed that individuals who are highly interested in oral care, prioritize it and do not perceive it as a burden [46]. Furthermore, the students’ perception of the knowledge acquired over the four years of their nursing education is relatively low.

On the other hand, it is noteworthy that nursing students demonstrate a high perception regarding both the significance of oral health in overall well-being and the role of nurses in promoting and preventing oral health. The fact that students with relatives working in the health field placed a higher value on the importance of oral health in overall health suggests that being in contact with individuals sensitized to the subject may influence this perception positively. In this context, activities like World Oral Health Day, proposed by the WHO, could play a crucial role in raising awareness about the significance of oral health [47].

Additionally, their motivation to enhance their knowledge in this field is also high, as evidenced by their desire to incorporate more oral health content into the nursing curriculum. These results demonstrate the sensitivity of nurses in oral health, which is positive since according to Ashour [48], it is essential to provide healthcare workers with training on the importance of oral health and consider it a patient’s right rather than a privilege.

Therefore, there is a need to improve the oral health’s knowledge of the future nurses and in this sense, an interdisciplinary cooperation between nursing and dental professionals for the development of an oral health curriculum for nurses, which could promote and improve oral health and prevent dental diseases in the community (16). Moreover, our results suggest that employing problem-based learning methodologies could serve as a viable strategy to enhance oral health knowledge among nursing students.

### 4.1. Implication for Practice

According to the World Health Organization, oral diseases, while largely preventable, pose a major health burden for many countries and affect people throughout their lifetime, causing pain, discomfort, disfigurement, and even death [2]. Moreover, nurses must have an important role in promoting oral health. Possessing an awareness on the oral health knowledge of nursing students is important for the universities and health managements to implement new strategies in order to acquire better nursing competencies in these fields. Improving nursing students’ oral health knowledge will make them better equipped to provide comprehensive community oral care, including preventive measures and the early identification of oral health issues. This can significantly contribute to the overall well-being of the person as oral health is intricately linked to general health.

In addition, this is the first study to describe the situation of nursing students in the University of Girona with the purpose to evaluate future strategies aimed at increasing the knowledge of nursing students in oral health. In this line, we acknowledge the significance of having a validated questionnaire, which should be pursued as a separate research line. The validation of a questionnaire to assess the knowledge of nursing students in oral health could prove invaluable in monitoring the evolution of this knowledge among them.

### 4.2. Limitations

The main limitation identified in this study was the use of a cross-sectional design, which only allowed to study the relationship between variables without the possibility of establishing causality, even though the calculated sample size was appropriate. In addition, the results only reflected these students’ knowledge at the time of the study, which has limitations when making future projections.

Another limitation is the lack of a validated questionnaire to assess the knowledge of nursing students in oral health. However, we addressed this issue by developing a questionnaire in collaboration with experts in the field and by consulting the most recent literature. The questionnaire demonstrated a good internal consistency. The fact that students completed the questionnaire without any supervision could have also led to the potential copying of answers. Therefore, the time to complete the questionnaire was limited to 10 min. Finally, despite the fact that the sample size was not very large, it was sufficient to comprehend the situation in the form of a first snapshot of nursing students in relation to oral health knowledge.

## 5. Conclusions

The study findings showed that most of the final-year nursing students attended regular dental check-ups for preventive purposes and half of them experienced cavities at some point in their lives. In addition, the students’ knowledge of oral health is moderate and their perception of the knowledge acquired over the four years of their nursing education in this field is relatively low. However, they are aware of the importance of oral health on the population’s overall well-being and the role of nurses in promoting and preventing oral health and are motivated to increase their knowledge if the nursing curriculum offers the opportunity while considering the use of problem-based learning methodology as the best way to increase this knowledge.

## Figures and Tables

**Table 1:** Sociodemographic and oral health characteristics of the sample.

	Total Study Population (*n*: 72)
Age (Mean, DS)	22.7 (2.8)
Gender	*n* (%)
Woman	61 (84.7)
Man	5 (6.9)
Non-binary	-
Place of Origin	
Catalonia	47 (65.3)
Spain	13 (18.1)
Outside of Spain	6 (8.3)
Previous Education	
Secondary Education	48 (66.7)
Training Cycle	10 (13.9)
University Degree	8 (11.1)
Employment	
Yes	45 (62.5)
No	21 (29.2)
Working in the healthcare field	
Yes	45 (62.5)
No	21 (29.2)
Employment Field	
Hospital	31 (43.1)
Primary Care	-
Nursing Homes	14 (19.5)
Family members working in health field	
Yes	34 (47.2)
No	32 (44.4)

Descriptive results: *n* (%). Frequency (percentage).

**Table 2:** Nursing students’ personal variables related to oral health.

	Total Study Population (*n*: 72)
Undergoing preventive dentist check-ups	*n* (%)
Yes	60 (83.3)
No	6 (8.3)
Frequency of dentist visits	
Less than once a year	25 (34.7)
Once a year	30 (41.7)
More than once a year	5 (6.9)
More than twice a year	6 (8.3)
Presence of cavities	
Yes	48 (66.7)
No	17 (23.6)
Number of cavities	
I don’t know	9 (12.5)
One	15 (20.8)
Between two and five	25 (34.7)
More than five	7 (9.7)
Carrier of Orthodontics	
Yes	39 (54.2)
No	27 (37.5)

Descriptive results: *n* (%). Frequency (percentage).

**Table 3:** Results of each question of the test.

	Total Study Population (*n*: 72)
General knowledge of oral health (Percentage of correct answers)	*n* (%)
Correct definition of oral health	44 (61.1)
Knowledge of the most prevalent oral pathology (cavities)	65 (90.3)
Knowledge of teething (Percentage of correct answers)	
Correct number of teeth sets throughout human lifetime	59 (81.9)
Regarding teething, choose the correct answer	41 (56.9)
Knowledge of dental tissue (Percentage of correct answers)	
Incorrect dental tissue identification	53 (73.6)
Knowledge about aspects of teeth (Percentage of correct answers)	
Knowledge of first teeth appearance	54 (75)
Knowledge of main teeth functions	21 (29.2)
Knowledge of involuntary teeth grinding	23 (31.9)
Knowledge of oral health problems’ risk factors and preventive activities (Percentage of correct answers)	
Correct identification of gingivitis risk factors	9 (12.5)
Correct identification of cavities risk factors	48 (66.7)
Correct identification of agenesis risk factors	58 (81.9)
Correct identification of periodontitis risk factors	54 (75.0)
Correct identification of the use of the Modified Bass technique	23 (31.9)
Correct identification of the amount of toothpaste	17 (23.6)
Correct identification of the predictable oral diseases risk factors	39 (54.2)
Correct identification of the most prevalent oral pathologies	34 (47.2)

Descriptive results: *n* (%). Frequency (percentage).

**Table 4:** Participants’ perception of the importance of oral health and the knowledge acquired during the nursing degree.

	Total Study Population (*n*: 72) (Mean, DS)
Importance of oral health in overall health	4.5 (0.6)
Importance of the nursing role in the prevention and promotion of oral health	4.2 (0.9)
Observation of prevention and promotion activities related to oral health during clinical practices	2.8 (1.2)
Acquisition of oral health knowledge during the nursing degree	2.5 (0.9)
Satisfaction with the oral health content received during the nursing degree	2.5 (0.8)
Satisfaction with the oral health content received through master classes	2.4 (0.9)
Satisfaction with the oral health content received through seminars	2.3 (0.9)
Satisfaction with the oral health content received through problem-based learning methodology	2.7 (0.4)
Consideration of the need to receive more oral health content in the nursing degree	4.4(0.6)

Descriptive results: mean (standard deviation).

## Data Availability

The data presented in this study are available on request from the corresponding author.

## Public Involvement Statement

No public involvement in any aspect of this research.

## Guidelines and Standards Statement

This manuscript was drafted against the STROBE [49] guidelines for observational studies.
